# Dynamic expression of cytokine and transcription factor genes during experimental *Fasciola gigantica* infection in buffaloes

**DOI:** 10.1186/s13071-017-2538-1

**Published:** 2017-12-08

**Authors:** Wei Shi, Zhi-Yong Wei, Hany M. Elsheikha, Fu-Kai Zhang, Zhao-An Sheng, Ke-Jing Lu, Dong-Ying Wang, Wei-Yi Huang, Xing-Quan Zhu

**Affiliations:** 1 College of Animal Science and Technology, Guangxi University, Nanning, 530005 Guangxi Zhuang Autonomous Region People’s Republic of China; 2 State Key Laboratory of Veterinary Etiological Biology, Key Laboratory of Veterinary Parasitology of Gansu Province, Lanzhou Veterinary Research Institute, Chinese Academy of Agricultural Sciences, Lanzhou, 730046 Gansu Province People’s Republic of China; 3 Faculty of Medicine and Health Sciences, School of Veterinary Medicine and Science, University of Nottingham, Sutton Bonington Campus, Loughborough, LE12 5RD UK; 4 Jiangsu Co-innovation Center for the Prevention and Control of Important Animal Infectious Diseases and Zoonoses, Yangzhou University College of Veterinary Medicine, Yangzhou, Jiangsu Province, 225009 People’s Republic of China

**Keywords:** *Fasciola gigantica*, Buffaloes, Liver, Cytokines, Transcription factors, Gene expression

## Abstract

**Background:**

Determining the mechanisms involved in the immune-pathogenesis of the tropical liver fluke, *Fasciola gigantica*, is crucial to the development of any effective therapeutic intervention. Here, we examined the differential gene expression of cytokines and transcription factors in the liver of *F. gigantica*-infected buffaloes, over the course of infection.

**Methods:**

Water buffaloes (swamp type) were infected orally with 500 *F. gigantica* encysted metacercariae. Liver tissue samples were collected 3, 10, 28, 42, 70 and 98 days post-infection (dpi). Levels of gene expression of nine cytokines (IFN-γ, TGF-β, IL-1β, IL-4, IL-6, IL-10, IL-12B, IL-13 and IL-17A) and four transcription factors (T-bet, GATA-3, Foxp3 and ROR-γτ) were determined using quantitative real-time PCR (qRT-PCR). We evaluated any correlation between gene expression of these immune-regulatory factors and the severity of liver pathology.

**Results:**

Histopathological examination revealed that cellular infiltration, hemorrhage and fibrosis without calcification in the liver parenchyma of infected buffaloes, increased over the course of infection. This progressive pathology was attributed to dysregulated and excessive inflammatory responses induced by infection. The early infection phase (3–10 dpi) was marked by a generalized immunosuppression and elevated TGF-β expression in order to facilitate parasite colonization. A mixed Th1/Th2 immune response was dominant from 28 to 70 dpi, to promote parasite survival while minimizing host tissue damage. During late infection (98 dpi), the response was biased towards Th1/Treg in order to inhibit the host’s Th2 protective response and promote chronic infection. Both IL-10 and IL-17A and the Th17/Treg balance, played key roles in mediating the inflammatory and immunoregulatory mechanisms in the liver during chronic fasciolosis.

**Conclusions:**

Our data showed distinct CD4^+^ T helper (Th) polarization and cytokine dysregulation in response to *F. gigantica* infection in water buffaloes over the course of infection. Characterizing the temporal expression profiles for host immune genes during infection should provide important information for defining how *F. gigantica* adapts and survives in the liver of buffaloes and how host immune responses influence *F. gigantica* pathogenicity.

## Background

Fasciolosis is a zoonotic parasitic disease caused by infection with the digenetic trematode flukes of the genus *Fasciola*. While *Fasciola hepatica* is prevalent in temperate regions, *F. gigantica* is more widespread in Africa and Asia [1, 2]. Migration of these flukes inside the body of the host causes severe damage to the liver parenchyma and gall-bladder [3–5]. Buffaloes are economically important animals for the farming communities in developing countries. Infection of buffaloes with *F. gigantica* is common in southern China and other geographic regions of the world [6]. Infection can cause poor animal health and significant loss of meat and milk production, with considerable financial implications [3, 7]. *Fasciola gigantica* flukes specifically target the liver of their definitive host. Effective and balanced local immunity is therefore essential for detecting and controlling these hepatotropic parasites, and for limiting hepatic damage.

Liver flukes are, however, efficient immune-modulators and produce many effectors in order to exploit the host immune response to ensure their survival. A recent study in experimentally infected buffaloes reported a modest increase in the level of Th2-type immune cytokines during early *F. gigantica* colonization and immunosuppression during chronic *F. gigantica* infection [8]. Other studies reported a pro-inflammatory or a mixed Th1/Th2 immune response during early infection, and heightened Th2 and Treg responses during chronic infection. This heightened response was assumed to play roles in restoring the host tissue integrity by damping excessive inflammatory response [9, 10]. How inflammation contributes to the pathogenesis of *F. gigantica* is a complex and multi-faceted story that is still unfolding.

Hepatic immune-inflammatory mechanisms are essential to maintain liver homeostasis and, if dysregulated (e.g. due to parasite infection), can lead to liver pathology and dysfunction. The abnormal production of cytokines and/or transcription factors can lead to inadequate control of *Fasciola* infection [11, 12]. CD4^+^ T-cells are subdivided into Th1, Th2, Th17 and regulatory T-cells (Treg) subsets, based on their pattern of cytokine production [13]. Transcription factors T-bet, GATA-3, Foxp3 and ROR-γτ play important roles in the differentiation of Th1, Th2, Treg and Th17 cells respectively, and mediate the production of cytokines in these cells [14–16]. Although immunological impairment and polarization of the Th1/Th2 balance are major consequences of *F. gigantica*-induced liver pathology, the expression profile and dynamic changes of Th1/Th2 cytokines during *F. gigantica* infection has not been completely elucidated. Also, the role of Th17 and Treg cells in the pathogenesis of *F. gigantica* infection is still not well-defined.

In the present study, we hypothesized that *F. gigantica* infection impairs the balance of Th subsets (Th1/Th2/Th17) and Treg, thus contributing to the immune-pathogenesis of fasciolosis. A temporal study of gene expression of nine cytokines (IFN-γ, IL-1β, IL-12B, IL-4, IL-6, IL-10, IL-13, IL-17A and TGF-β) and four transcription factors (T-bet, GATA-3, Foxp3 and ROR-γτ) in the livers from buffaloes infected with *F. gigantica* was conducted using quantitative real-time PCR (qRT-PCR). Data analyses revealed a large number of differentially regulated genes, which exhibited temporal profiles of expression across the time course study. Our results showed evidence of a step-change in gene expression from an ‘early’ TGF-β-associated immune-suppersive response (3–10 dpi), to a mixed Th1/Th2 immune response (28–70 dpi) and a ‘late’ predominantly Th1/Treg-driven response (98 dpi). These results provide new insights into the dynamic immune response of buffaloes to *F. gigantica* over the course of experimental infection.

## Methods

### Parasite strain

Eggs of *F. gigantica* were obtained from the gall bladder and faeces of naturally infected buffaloes slaughtered for human consumption at local abattoirs (Nanning, Guangxi, P.R. China). Protocols used for the preparation of *F. gigantica* eggs, snail infection with miracidia and harvesting of encysted metacercariae (EM), were performed as previously described [16]. EM were stored in sterile phosphate buffered solution (PBS) at 4 °C. We employed PCR amplification and sequencing of the second internal transcribed spacer (ITS-2) of ribosomal DNA (rDNA) to genotype EM, as described previously [17]. Species identity was confirmed as *F. gigantica* based on absolute homology to the known ITS-2 sequence of *F. gigantica* from Guangxi province (GenBank: AJ557569). The viability of EM was examined microscopically and only those with viability greater than 90% were used.

## Animals

Eight to ten month-old (80–100 kg body weight) water buffaloes (*n* = 35) were obtained from a local breeder and were identified as swamp type by karyotypic analysis. Buffaloes were kept in separate concrete floor pens. Commercial feed and clean water were provided ad libitum for all animals during the entire study period. None of the buffaloes had been used previously for any experimental procedure. Animals were confirmed as negative in terms of prior infection with liver flukes, by negative fecal examination and negative serum *F. gigantica*-specific IgG-antibody-based ELISA prior to the start of the study. All animals were treated with a single dose of triclabendazole (5% *w*/*v*) in order to eliminate any potential existing fluke infection that may have been missed on laboratory examination. Following triclabendazole treatment, buffaloes were allowed to acclimatize for 30 days to avoid any residual efficacy of the treatment on the establishment of experimental *F. gigantica* infection.

### Animal inoculation and tissue collection

Thirty-five buffaloes were assigned randomly to seven different groups (5 buffaloes/group). Group I was composed of 5 buffaloes that were mock-incoulated with PBS only. Buffaloes in Groups II-VII were each infected with 500 viable metacercariae by oral gavage. Control buffaloes were euthanized at the start of the experiment to obtain baseline values for hepatic tissue pathology and gene expression. Animals from each of the six infected groups were sacrificed and their livers were harvested at 3, 10, 28, 42, 70 and 98 days post infection (dpi), for histopathological and molecular studies. Group III-VII buffaloes were examined clinically on a weekly basis for the development of clinical signs of fasciolosis. The control group served as a baseline point of reference for monitoring the progressive changes in gene expression over the course of infection. However, the inclusion of matched control groups receiving placebo (treated with vehicle only) and euthanized at the same time points as infected groups would have strengthened the power of the study. Alternatively, control buffaloes could have been kept alive (rather than killing them at the start) and liver punch biopsy samples obtained from them at each of the above time points. Despite the early sacrifice of the control animals for reasons of economy and resources, we believe the effects we have observed to be due to the experimental manipulation.

### Gross examination and histopathological evaluation

At six time points after infection (indicated above), animals in each infected group were sacrificed, their livers were harvested and examined for pathological lesions and the presence of the flukes. Parasite eggs were recovered by filtering bile fluid through a 0.15 mm pore size mesh. *Fasciola gigantica* infection was confirmed by observing gross pathological lesions, associated with flukes in the livers and/or by the presence of flukes and eggs in the bile ducts. Samples of liver tissue (~8 g) showing pathological lesions were collected from each animal. Tissue samples were resuspended in 10% PBS-buffered formalin solution overnight, then dehydrated in alcohol, rinsed in xylene, and embedded in paraffin. 3 μm sections of paraffin-embedded tissue were mounted onto glass slides, and stained with hematoxylin and eosin (H&E). Stained tissue sections were examined microscopically at 400× magnification and imaged using a Zeiss Axio Imager manual upright research microscope. Additional liver tissue samples for RNA extraction were collected and kept in RNA store buffer (Tiangen Biotech, Beijing, China), snap frozen in liquid nitrogen and stored at -80 °C.

### RNA isolation

Total RNA was extracted from frozen liver tissue samples by RNAprep Pure Tissue Kit (Tiangen Biotech, Beijing, China) following the manufacturer’s instructions. RNA integrity was examined by 2% agarose gel electrophoresis and quantified by NanoDrop 2000/2000c Spectrophotometer analysis (Thermo Scientific, Waltham, US).

### Quantification of cytokine and transcription factor gene expression

Quantitative gene expression analysis was performed on liver samples obtained from uninfected control animals, and from infected animals on 3, 10, 28, 42, 70 and 98 dpi. Levels of mRNA expression of nine cytokines (IFN-γ, IL-1β, IL-12B, IL-4, IL-6, IL-10, IL-13, IL-17A and TGF-β) and four transcription factors (T-bet, GATA-3, Foxp3 and ROR-γτ) were determined using quantitative real-time PCR (qRT-PCR). All qRT-PCR primers used in the study are described in Table 1. Complementary DNA (cDNA) was synthesized from 500 ng RNA samples using a PrimerScript™ RT reagent kit (TaKaRa Bio, Dalian, China). qRT-PCR was performed using SYBR®Premix Ex Taq™ II (Tli RNaseH Plus, TaKaRa Bio) and a CFX96 real-time PCR instrument (Bio-Rad, Hercules, US). To determine the specificity of amplification, melting curve analysis was applied to all final PCR products. The efficiency of qRT-PCR, and relative quantification (*RQ*) of gene expression, were analyzed using the comparative 2^–ΔΔ*Cq*^ method [18]. The level of expression of each gene was normalized using glyceraldehyde-3-phosphate dehydrogenase (GAPDH) as the reference housekeeping gene.

**Table 1:** List of primers used in the SYBR green-based qRT-PCR analysis

Gene target		Primer sequence (5′–3′)	Product length (bp)	Reference
GAPDH	F	CCTGCACCACCAACTGCTTG	222	[62]
	R	TTGAGCTCAGGGATGACCTTG		
IFN-γ	F	GTCTCCTTCTACTTCAAACT	253	[63]
	R	ATTCTGACTTCTCTTCCGCT		
TGF-β	F	CGTGCTAATGGTGGAATAC	208	Present study
	R	GCCAGGAATTGTTGCTATA		
IL-1β	F	CTAGCCCATGTGTGCTGAAG	59	[62]
	R	CCTTTACTTGGCTCTTCACC		
IL-4	F	CAGCATGGAGCTGCCT	177	[64]
	R	ACAGAACAGGTCTTGCTTGC		
IL-6	F	CTGCAATGAGAAAGGAGATA	191	[63]
	R	GGTAGTCCAGGTATATCTGA		
IL-10	F	CTGTGCCTCTCCCCTAGAGT	236	[62]
	R	GCAGCTAGCTCCACAAGGAA		
IL-12B	F	CAGGGACATCATCAAACCAG	213	[63]
	R	CTTGTGGCATGT GACTTTGG		
IL-13	F	AGAACCAGAAGGTGCCGCT	50	[65]
	R	GGTTGAGGCTCCACACCATG		
IL-17A	F	CTACAGTGAACTGGAAGGAAC	554	Present study
	R	AAAAGGGGCTGGGTCT		
T-bet	F	CCTGGACCCAACTGTCAACT	171	[66]
	R	GAAACTCGGCCTCATAGCTG		
GATA-3	F	GATCAAGCCCAAGCGAAGG	124	Present study
	R	CCGCAGGCATTGCAGACA		
Foxp3	F	GACAGCACCCTTTCGACTGT	191	[66]
	R	CTCCAGAGATTGCACCACCT		
ROR-γτ	F	CTACAGTGAACTGGAAGGAAC	554	Present study
	R	AAAAGGGGCTGGGTCT		

*Abbreviations*: *F* forward primer, *R* reverse primer

### Data analysis

Statistical analysis and graph production were performed using GraphPad Prism (GraphPad Software Inc., La Jolla, CA, USA, version 6.02.). Levels of cytokine and transcription factor mRNA expression between uninfected and infected groups were compared at different time points after infection using one-way analysis of variance (ANOVA) with *post-hoc* LSD multiple comparison tests. Results were presented as *F*_(DFn, DFd)_ and *P*-value. Pearson’s correlation coefficients (*r*-value) were used to detect any correlation between the measured level of Th1, Th2, Treg and Th17 immune cytokines and transcription factors (T-bet, GATA-3, Foxp3 and ROR-γτ) gene expression between infected groups, followed by a two-tailed *post-hoc* test and presented as a *P-*value. Data shown represent the mean ± SEM of results from five buffaloes. The level of significance for all analyses was evaluated with a confidence interval > 95% (*P* < 0.05).

## Results

### Gross and histopathological attributes

**Fig. 1:**
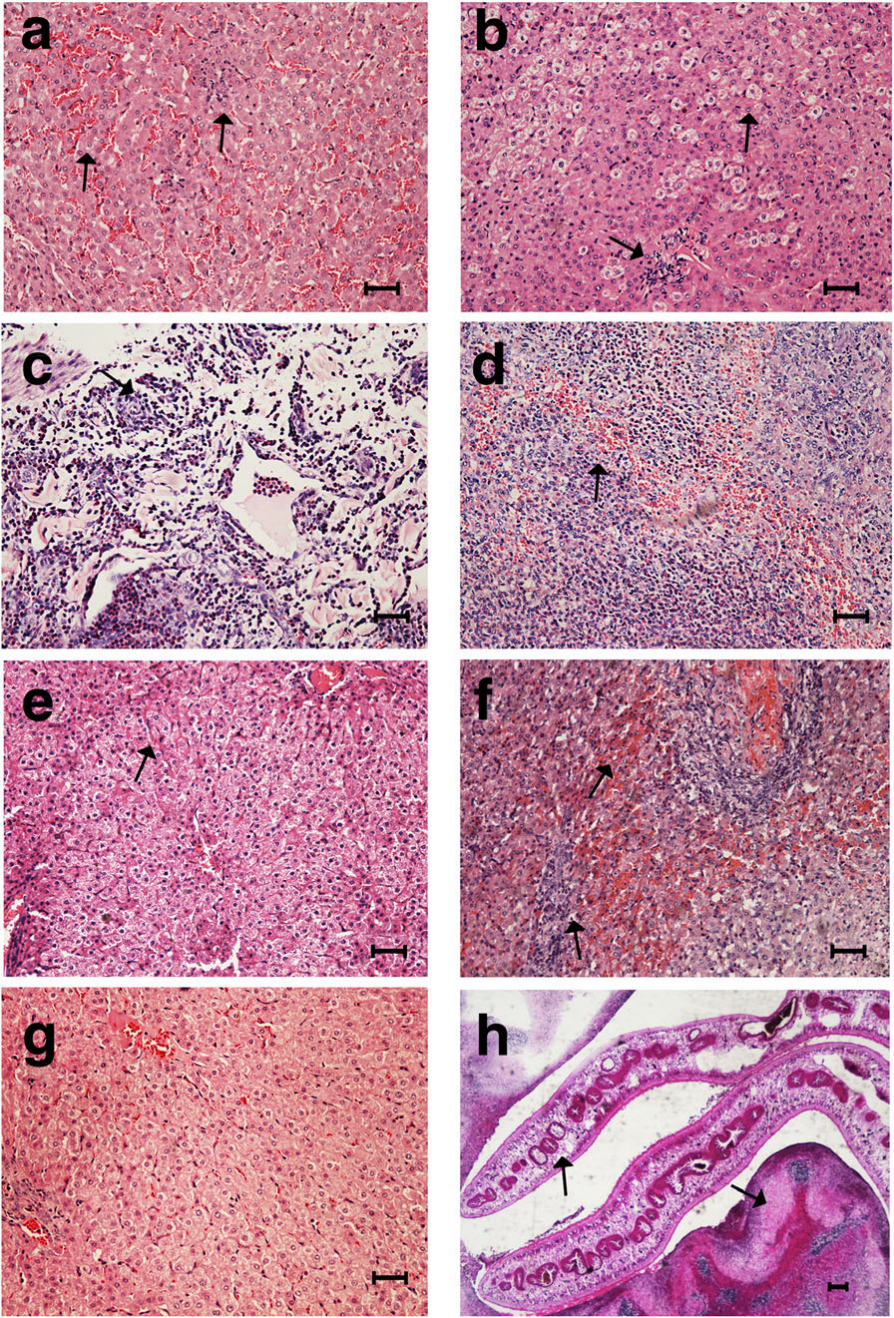
Histopathological characteristics of the livers of buffaloes infected with *Fasciola gigantica*. **a** At 3 dpi, there was local hyperemia associated with mild filtration of lymphocytes and neutrophils. **b** At 10 dpi, there was scattered vacuolation of hepatocytes, consistent with fat, along with mild to moderate focal necrosis. **c** At 28 dpi, diffuse intravascular coagulation, severe infiltration of inflammatory cells mainly neutrophils and lymphocytes, and granular degeneration of the cytoplasm were observed. **d** At 42 dpi, moderate to severe multifocal hemorrhages and necrosis, infiltration of eosinophils, RBCs and monocytes, and accumulation of fibroblasts were detected. **e** At 70 dpi, there were mild to moderate multifocal bile duct hyperplasia and focal coagulative necrosis associated with collagen deposition. **f** At 98 dpi, severe periportal fibrosis associated with multifocal inflammatory infiltrate, cellular debris, moderate multifocal hemorrhages, and granulomas with necrotic centres were detected. **g** Liver tissue from uninfected animal showed normal histological architecture of the hepatic tissue. **h** Adult flukes in the intrahepatic bile duct, along with epithelial hyperplasia of the duct. In all figures, tissue sections were stained with H&E and arrows point at the corresponding morphological features described above. *Scale-bars*: **a-g**, 50 μm; **h**, 100 μm

Even though buffaloes did not exhibit clear clinical signs, *F. gigantica* induced a wide range of pathological lesions over the course of infection (Table 2). During the early stage of infection (3–70 dpi), immature flukes migrated through the intestinal wall, abdominal cavity, liver capsule and liver parenchyma, to reach the bile ducts where they attain sexual maturity. Migration of the juvenile flukes through the host’s tissues was characterized by classical inflammatory signs and accumulation of fibroblasts towards the end of this stage, but without obvious fibrosis. The total number (and average length) of flukes recovered from the livers of infected groups at 28, 42, 70 and 98 dpi were: 3 flukes (1.5 mm), 22 flukes (2.5 mm), 65 flukes (8 mm), and 36 flukes (14 mm), respectively. No fluke was observed in the liver or bile duct before 28 dpi, probably because they were too small to be visible to the naked-eye. Hepatic hemorrhage, swelling, necrosis and viscous bile were first detected at 10 dpi. Tissue fibrosis was observed at 70 dpi, along with disappearance of hemorrhage. Seven parameters were used to describe the histopathological features in buffalo livers during infection (Table 3). Histopathological characteristics of the infected liver tissues (Fig. 1) included an infiltration of inflammatory cells, such as neutrophils and lymphocytes at 3 dpi. As infection progressed, an accumulation of eosinophils, monocytes and red blood cells (RBCs) was detected (10–28 dpi), while fibroblast formation and bile duct hyperplasia were observed later (42 dpi). During the chronic stage (70–98 dpi), significant fibrosis and necrosis, without calcification, were observed. Fluke eggs were detected in the bile and feces of infected animals at 98 dpi. Granulomas with necrotic centers, heavy infiltrates of lymphocytes, RBCs and eosinophils were present at 98 dpi.

**Table 2:** The presence (+) and absence (−) of gross pathological lesions, flukes and fluke eggs in the liver of buffaloes experimentally infected with *F. gigantica*

Group	Dpi	No. of animals	Hemorrhage	Swelling	Parasite tunnel	Fibrosis	Necrosis	Bile duct hyperplasia	Viscous bile	Visible flukes	Eggs
I	0	5	–	–	–	–	–		–	–	–
II	3	5	–	–	–	–	–	–	–	–	–
III	10	5	+	+	–	–	+	–	+	–	–
IV	28	5	+	+	–	–	+	–	+	+	–
V	42	5	–	+	+	–	+	+	+	+	–
VI	70	5	–	+	+	+	+	+	+	+	–
VII	98	4^a^	–	+	+	+	+	+	+	+	+

^a^RNA from one animal in this group VII did not pass the quality control check and was therefore excluded from the analysis

**Table 3:** The presence (+) and absence (−) of the histopathological changes observed in the liver of buffaloes experimentally infected with *F. gigantica*

Group	Dpi	No. of animals	Inflammatory infiltration	RBCs	Eosinophils	Neutrophils	Fibroblasts	Lymphocytes	Monocytes
I	0	5	–	–	–	–	–	–	–
II	3	5	+	–	–	+	–	+	–
III	10	5	+	+	+	+	–	+	–
IV	28	5	+	+	+	+	–	+	+
V	42	5	+	+	+	+	+	+	+
VI	70	5	+	+	+	+	+	+	+
VII	98	4	+	+	+	+	+	+	+

### Gene expression of cytokines

**Fig. 2:**
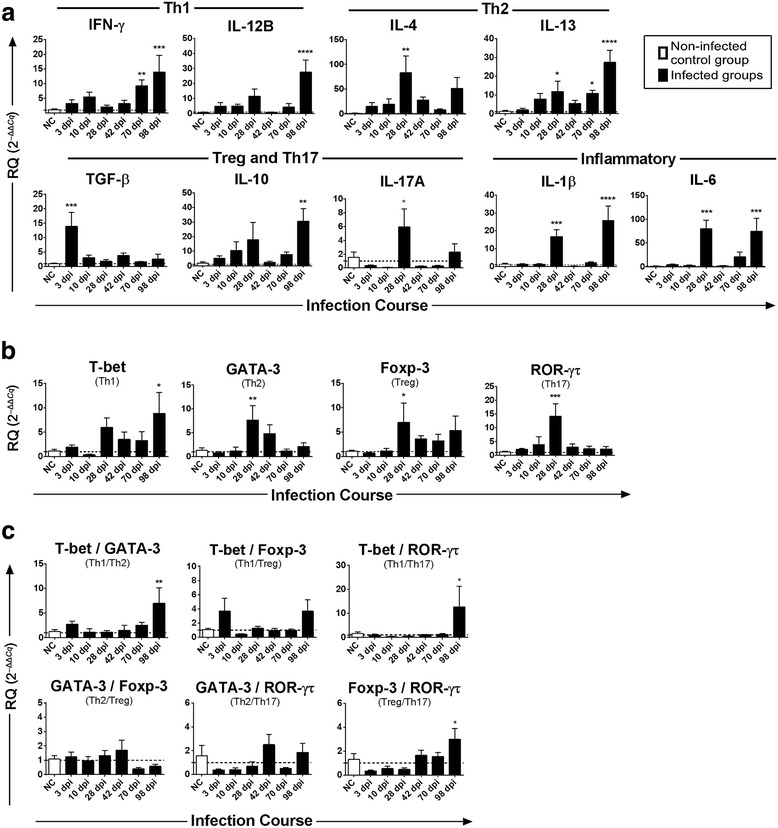
Temporal changes of the mRNA expression of cytokines and transcription factors in the liver of buffaloes experimentally infected with *Fasciola gigantica*. The X axis represents days post infection (dpi), control animals are represented by empty bars and Y axis represents the mRNA relative expression of target genes relative to *GAPDH* based on 2^–ΔΔ*Cq*^ calculation. Columns show the means and error bars show SEMs. The log number of mRNA relative expression of **a** IFN-γ, IL-1β, IL-4, IL-6, IL-10, IL-12B, IL-13, IL-17A, TGF-β and **b** T-bet, GATA-3, Foxp3, and ROR-γτ, are shown. **c** Trends in the ratios between each pair of transcription factors during the course of infection, which was used as indicators of the balance between Th1/Th2 and Treg/Th17. Significant differences of each time-point compared with control non-infected (NC) group: *P < 0.05; **P < 0.01; ***P < 0.001 or ****P < 0.0001 (analyzed by one-way ANOVA, *post-hoc* LSD test)

To determine how immune response changed over the course of infection, gene expression of nine cytokines was assessed in the liver tissue of 29 infected buffaloes and five uninfected buffaloes using qRT-PCR. RNA from one animal from group VII did not pass the quality control check and was therefore excluded from the analysis. Results showed that infection had a significant impact on gene expression of Th1, Th2, Th17, and Treg cytokines (Fig. 2a and Table 4). The transcriptional profile of cytokine genes showed an immunosuppressive state during early infection, a mixed Th1/Th2 response as the infection progressed, and a shifting to Th1/Treg response, associated with greater histopathological changes and fibrosis during late stage infection.

**Table 4:** Relative gene expression in the liver of buffaloes experimentally infected with *F. gigantica* compared to the control group. The relative quantities were obtained according to the comparative *Cq* method (2^–ΔΔ*Cq*^). *P* values indicate statistical significance (*P* < 0.05)

Gene Target	ANOVA		Multiple comparisons											
			3 dpi		10 dpi		28 dpi		42 dpi		70 dpi		98 dpi	
	*F*_(DFn, DFd)_	*P*	*RQ*	*P*	*RQ*	*P*	*RQ*	*P*	*RQ*	*P*	*RQ*	*P*	*RQ*	*P*
IFN-γ	4.693_(6, 21)_	0.0035	2.08	0.4326	5.51	0.1282	2.06	0.7358	3.21	0.4590	9.27	0.0079	13.91	0.0003
TGF-β	4.738_(6, 19)_	0.0041	13.93	0.0003	3.07	0.4884	1.67	0.8036	3.85	0.3408	1.63	0.8371	2.66	0.6062
IL-1β	11.05_(6, 26)_	< 0.0001	1.31	0.9767	1.27	0.9811	14.25	0.0007	0.26	0.8137	2.31	0.8025	28.45	< 0.0001
IL-4	2.877_(6, 24)_	0.0291	7.01	0.5847	19.4	0.4892	97.21	0.0028	27.9	0.3121	9.43	0.7749	67.14	0.0630
IL-6	7.636_(6, 24)_	0.0001	5.50	0.8529	2.81	0.9366	78.1	0.0002	1.94	0.9728	23.14	0.2805	94.56	0.0008
IL-10	2.514_(6, 26)_	0.0471	5.99	0.7003	9.92	0.3184	21.18	0.0694	2.3	0.9579	6.24	0.4902	32.67	0.0036
IL-12B	5.595_(6, 26)_	0.0008	5.92	0.4786	3.99	0.4746	13.68	0.0759	0.86	0.9825	5.37	0.5312	24.84	< 0.0001
IL-13	6.56_(6, 20)_	0.0006	2.09	0.8459	7.80	0.1522	11.87	0.0259	5.45	0.3499	10.8	0.0426	27.33	< 0.0001
IL-17A	3.532_(6, 21)_	0.0142	0.33	0.4612	0.07	0.3694	5.95	0.0114	0.26	0.4327	0.28	0.4400	2.99	0.6354
T-bet	2.173_(6, 28)_	0.076	2.16	0.7923	0.25	0.7830	4.73	0.1018	3.93	0.4158	3.99	0.4606	11.38	0.0113
GATA-3	3.319_(6, 28)_	0.0135	0.84	0.8101	1.42	0.9579	5.18	0.0035	4.80	0.0889	1.34	0.9397	1.8	0.6952
Foxp3	1.455_(6, 28)_	0.2296	0.70	0.9054	1.07	0.9749	3.03	0.0424	3.97	0.3705	3.40	0.4458	3.05	0.1372
ROR-γτ	4.387_(6, 28)_	0.003	2.25	0.7567	4.57	0.3780	10.28	0.0002	3.24	0.5637	2.68	0.7076	2.55	0.7148

*Abbreviations*: *RQ*, relative quantity (mean); *F*_(DFn, DFd)_, degrees of freedom with numerator and degrees of freedom denominator inside the subscript parentheses

The gene expression of Th1 cytokines (IFN-γ and IL-12B) and IL-10 showed no significant change during early infection. IFN-γ showed significantly higher expression at 70 dpi (*P* = 0.0079) and 98 dpi (*P* = 0.0003), when compared with the control. Relative gene expression of both of IL-12B (*P* < 0.0001) and IL-10 (*P* = 0.0036) peaked at 98 dpi. Fluke infection also upregulated IL-4 expression at 28 dpi (*P* = 0.0028), whereas IL-13 significantly increased at 28 dpi (*P* = 0.0259) and 70 dpi (*P* = 0.0426), peaking at 98 dpi (*P* < 0.0001). TGF-β mRNA was rapidly elevated at 3 dpi (*P* = 0.0003), followed by a decrease to the basal level and remained low thereafter. Upregulation of IL-17A was noted at 28 dpi (*P* = 0.0114), however expression was relatively low based on a high *Cq* value, suggesting it to be of little diagnostic value as a marker for Th17 cytokine in this study. The kinetics of the cytokines IL-1β and IL-6 mRNA were similar, both peaking at 28 dpi (*P* = 0.0007 for IL-1β; *P* = 0.0002 for IL-6) and 98 dpi (*P* < 0.0001 for IL-1β; *P* = 0.0008 for IL-6).

**Fig. 3:**
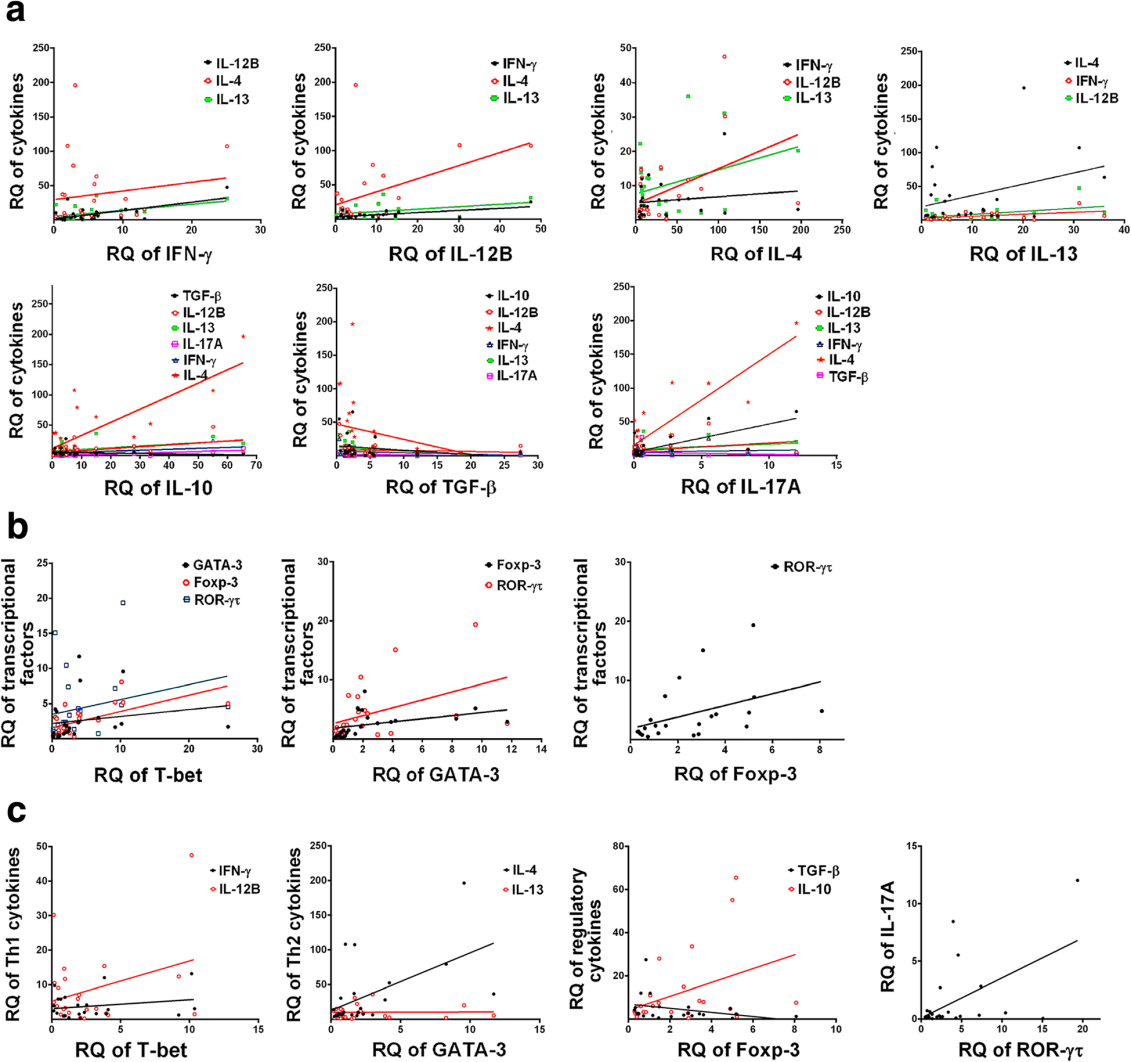
Pearson’s correlation analysis between mRNA expressions of Th1/Th2/Th17/Treg type **a** cytokines, **b** transcription factors, and **c** cytokines vs transcription factors in the liver of buffaloes infected with *Fasciola gigantica* (tested samples, *n* = 29) by pairwise comparison. Connecting lines illustrate positive (*r* > 0) and negative (*r* < 0) correlation indices

Spearman’s correlation analysis between each pair of cytokines was performed based on the *RQ* values from infected groups (Fig. 3a). This analysis revealed a significant positive correlation between the expression of IL-12B mRNA and Th2 cytokines (IL-4: *r*_(29)_ = 0.44, *P* = 0.0336; IL-13: *r*_(29)_ = 0.41, *P* = 0.0542), and between IFN-γ and IL-12B (*r*_(29)_ = 0.62, *P* = 0.0017) or IL-13 (*r*_(29)_ = 0.51, *P* = 0.0124). The gene expression of IL-10 was significantly correlated with both Th1 and Th2 cytokines (IFN-γ: *r*_(29)_ = 0.49, *P* = 0.0171; IL-12B: *r*_(29)_ = 0.50, *P* = 0.014; IL-4: *r*_(29)_ = 0.80, *P* < 0.0001; IL-13: *r*_(29)_ = 0.51, *P* = 0.014) and with IL-17A (*r*_(29)_ = 0.73, *P* < 0.0001). Positive correlation was also found between IL-4 and IL-13 (*r*_(29)_ = 0.44, *P* = 0.0336) gene expression. There was only a statistically significant relationship between IL-17A expression and IL-4 *r*_(29)_ = 0.87, *P* < 0.0001) and IL-10 (*r*_(29)_ = 0.73, *P* < 0.0001), but not with any of the other cytokines. Although not significant, the relative mRNA expression of TGF-β showed an inverse relationship to any other cytokine.

### Expression profiles of transcription factors

*Fasciola gigantica* infection altered the expression of T-bet, GATA-3 and ROR-γτ, with the expression of Foxp3 being least affected (Fig. 2b and Table 4). Compared with the control, mRNA of T-bet was highly expressed at 98 dpi (*P* = 0.0113), while the expression of the GATA-3, Foxp3 and ROR-γτ genes was upregulated at 28 dpi (*P* = 0.0035, *P* = 0.0424 and *P* = 0.0002, respectively). Spearman’s correlation analysis indicated that the expression of T-bet and Foxp3 was correlated (*r*_(29)_ = 0.63, *P* = 0.0011). Positive correlation was also found between the expression of ROR-γτ and GATA-3 (*r*_(29)_ = 0.43, *P* = 0.039) or Foxp3 (*r*_(29)_ = 0.42, *P* = 0.0439) (Fig. 3b). As shown in Fig. 3c, the mRNA expression of GATA-3 correlated significantly with that of IL-4 (*r*_(29)_ = 0.54, *P* = 0.0072), but was less correlated with another Th2 cytokine IL-13 (*r*_(29)_ = 0.017, *P* = 0.9387), whereas, the expression of ROR-γτ was correlated with that of IL-17A (*r*_(29)_ = 0.54, *P* = 0.0072). There was a tendency of correlation between the expression of T-bet and Th1 cytokine IFN-γ (*r*_(29)_ = 0.22, *P* = 0.305) or IL-12B (*r*_(29)_ = 0.33, *P* = 0.1265) and between the expression of Foxp3 and IL-10 (*r*_(29)_ = 0.38, *P* = 0.0718).

### Th1/Th2/Th17/Treg balance

To better understand the temporal trends in gene expression of Th1, Th2, Th17 and Treg in the liver post-infection, the ratios between the expression of transcription factors were evaluated using the pairwise comparison method (Fig. 2c). The *RQ* ratio of T-bet/GATA-3 was employed as an indicator of Th1/Th2 cytokine gene expression pattern. This ratio was higher in the liver at 98 dpi when compared to the uninfected group (*P* = 0.0049). This analysis also revealed that Th1/Th17 (*P* = 0.0246) and Treg/Th17 (*P* = 0.0126) were both upregulated at 98 dpi, when compared with the samples tested at other time points (3–70 dpi). However, no significant change was found in either Th1/Treg, Th2/Treg, or Th2/Th17 expression ratios at any of the examined time points. These data suggest a predominance of the Th1 and Treg cellular immune response at 98 dpi.

## Discussion

Our findings provide an increased understanding of the immuno-inflammatory response of buffaloes to *F. gigantica* infection through examining the correlation between gene expression of nine cytokines and four transcription factors in Th1, Th2, Th17 and Treg cells, together with the liver pathology during early, mid and late stages of infection. We initially characterized gross and histopathological changes in the liver at six time points following infection compared to uninfected control. Although infected buffaloes did not exhibit clinical signs, they developed hepatic gross pathologies similar to what have been observed in other studies [15, 19]. Histopathological features also agreed with previous studies on *F. gigantica* [9, 20, 21] and *F. hepatica* [22]. An unexpected finding was that none of the examined liver from infected animals showed calcium deposits. The difference in liver calcification between our study and previous reports may relate to variations in the experimental conditions, such as using different infectious doses, different parasite strains, the permissibility of the host species, the duration of the experimental infection, and using single infection vs repeated infections [21–23].

Next, we have shown that buffaloes showed a substantial liver immune response subsequent to oral challenge with 500 *F. gigantica* encysted metacercariae. Certain genes exhibited shifting temporal expression patterns as the infection proceeded. Expression of cytokine and transcription factor genes were decreased at 3 and 10 dpi, suggesting a general immunosuppression state to allow parasite colonization. Cytokine reduction during the early stage of *F. gigantica* infection has been reported previously [24, 25]. A previous study has documented the downregulation of MHC-II related genes, as well as the suppression of the host pro-inflammatory (Th1) immune response during early *F. gigantica* infection [16]. This immunosuppression might be attributed to an increased expression of the immunosuppressive cytokine TGF-β. While TGF-β was upregulated at 3 dpi, Foxp3 (Treg transcription factor) was upregulated at a later stage of infection (28 dpi), indicating that Tregs (also known as Foxp3-expressing cells) play a more diverse role than causing early immunosuppression in regulating immune responses. Although increased TGF-β at 3 dpi may have promoted the generation of Foxp3-expressing Tregs, increased IL-6 at 28 dpi may have abrogated the suppressive function of Tregs [26] by antagonizing TGF-β-induced Treg generation and stimulating Treg differentiation to Th17, as reported previously [27].

The inflammatory cascades triggered by *F. gigantica* must be tightly coordinated in order to avoid severe liver pathology. Th2 cells mediate humoral responses via induction of IL-4, IL-5, IL-9, IL-10 and IL-13 cytokines, which are important for controlling extracellular parasites [28]. In agreement with this, our results at 28 dpi showed a significant increase in the expression of the anti-inflammatory Th2-associated cytokines (IL-4, IL-13) and transcription factor (GATA-3), indicating the induction of Th2 response in liver tissue, probably to limit the fluke development and to balance the increased levels of inflammatory cytokines IL-1β, IL-6 and IL-17A. This response was expected because IL-4 is known to have the greatest effect in inducing Th2 differentiation [29] and the GATA-3 transcription factor is a key regulator of Th2 differentiation [30]. IL-4 stimulation may have activated STAT6, which is the major signal transducer in IL-4-mediated Th2 differentiation [31]. STAT6 is known to promote Th2 differentiation by stimulating GATA-3 [32]. Therefore, this coordinated response may be mediated by the parasite and/or the host in order to promote parasite survival while minimizing host hepatic damage. Th2-predominant response together with the suppression of Th1/Th17 response has been reported previously [33–36]. Interestingly, some molecules secreted by *Fasciola* species can suppress the differentiation of Th17 cells independently of Th2 cells differentiation, by altering the function of dendritic cells [37].

Although CD4^+^ Th17 cells play a role in the control of a variety of parasitic infections, the role of IL-17 produced by CD4^+^ Th17 cells in immunity to *F. gignatica* has not been clearly defined [38, 39]. Our current and previous studies [8], demostrated that IL-17 may play a role in the inflammatory process during early *F. gigantica* infection. Th17 cells are a newly-identified class of effector T cell, which produces IL-17A and IL-17F. Th17 cells can contribute to resistance to many intracellular [40] and extracellular parasites [8]. In addition to controlling infection, IL-17 expression has been associated with inflammatory and allergic responses [41]. TGF-β and IL-6 act cooperatively and non-redundantly to promote Th17 activity [27]. In agreement with this, TGF-β was upregulated at 3 dpi, and both IL-6 and IL-17A were upregulated at 28 dpi. IL-6 was found to be important in suppressing Treg generation in order to promote Th17 differentiation as discussed above. The expression of retinoic acid-related orphan receptors ROR-γt, a key transcription factor in Th17 differentiation [42, 43], was positively correlated with the *RQ* level of IL-17A at 28 dpi.

At 70 and 98 dpi a mixed Th1/Th2 type profile, supported by co-dominance of IFN-γ, IL-1β, IL-12B, IL-6, IL-4 and IL-13 cytokines, was observed as consistent with the result obtained by Kumar [44]. The high expression of Th1 cytokines (IL-1β and IL-6), suggesting acute inflammatory activity, correlated with hemorrhage, necrosis and severe infiltration of inflammatory cells in the liver. Th2 cytokines, on the other hand, often lead to chronic inflammatory response by promoting fibrosis and tissue remodeling [45, 46]. The accumulation of fibroblasts during chronic infection suggests that Th2 cytokine-mediated tissue repair was taking place. This state of immune homeostasis is probably required for parasite persistence and was correlated with an increased number of flukes. The T-bet/GATA-3 ratio, a marker of Th1/Th2 [47], was not altered during acute infection, but was significantly increased when the infection became chronic, suggesting more bias towards a Th1-mediated inflammatory response during late infection. This observation contradicts previous studies in mice, cattle and buffaloes infected with *F. gigantica* that reported a mixed Th1/Th2 response with a predominance of a Th2-biased pattern [48, 49]. Other studies have reported a Th2 response in early infection and an increased Th0-type response during chronic *F. gigantica* infection in cattle [35] and immunosuppression during chronic *F. gigantica* infection in buffaloes [8]. These differing findings between studies may result from different experimental conditions.

A synergism between IL-1β and IL-17 has previously been reported [50]. In our study, a strong correlation was observed between IL-17A and IL-4 and IL-10, as well as between ROR-γτ and GATA-3 and Foxp3, implying that induction of Th17 was paralleled by induction of both Th2 and Treg. This refutes the antagonistic relationship previously reported between a polarized Th2/Treg immune response and suppression of Th1/Th17 cytokines [11, 51–53]. A gradual upregulation of IL-10 mRNA expression was observed during early infection, in agreement with our previous finding in buffalo’s serum [8]. IL-10 levels significantly increased at 98 dpi, which was also reported in a study on *F. hepatica*-infected sheep [54]. IL-10 is considered to be a regulatory cytokine rather than a Th2 type cytokine [55, 56]. High levels of IL-10 can suppress excessive inflammation induced by the parasite [57, 58]. In our study, elevated levels of IL-10 and the concurrent upregulation of Th1 and Th2 cytokine genes in the liver suggest that IL-10 was required for the Th2 response to *F. gigantica* infection, instead of serving an anti-inflammatory function. Our data also showed that the *RQ* level of Foxp3 increased at 28 dpi. This level remained relatively high over the course of infection, which may have contributed to the Th1/Th2 balance in order to enhance parasite survival in the liver and to protect the host from over-inflammation [59]. Foxp3-expressing Treg cells are critical to maintaining immune homeostasis by minimizing tissue pathology, while modulating host immune response against helminth infections [60, 61]. The monitoring of Th17 and Treg cell offers a promising new perspective on the pathogenesis of *F. gigantica* infection and deserves further exploration.

## Conclusions

This is the first study to characterize the correlation between the expression of Th1, Th2, Th17, and Treg cytokines and transcription factors with liver pathology in buffaloes, during the course of experimental *F. gigantica* infection. The expression patterns for the examined genes indicated that there were periods of differential regulation during *F. gigantica* infection, which may suggest either a mechanism of immune evasion based on modulation of transcription or a mechanism used by host tissue to limit the infection and tissue damage. Gene expression profiles showed a significant T-cell adaptive immune suppression between 3 and 10 dpi to facilitate parasite colonization and a more substantial transcript differential expression change (mixed Th1/Th2 immune response) from 28 to 70 dpi, which may contribute to the parasite survival while minimizing host tissue damage. During late infection (98 dpi), the response was biased towards Th1/Treg in order to limit the host’s Th2 protective response and promote chronic infection, which conincided with an increase in the number of recovered flukes and greater histopathological changes and fibrosis. Clearly, regulation of immune cytokine and transcription factor gene expression during *F. gigantica* infection is a complex mechanism involving a variety of parasite-specific and host-specific factors, perhaps depending on the parasite’s needs for survival during various phases of infection. Research into the mechanisms governing differential expression of these immune-related genes, may shed light on the actual role of these immune regulatory factors in the pathogenesis of *F. gigantica* infection in buffaloes. Much remains to be elucidated about the role of various types of inflammatory responses, and the cross-talk between types of T-helper responses, in fasciolosis.

## Abbreviations

ANOVA: Analysis of variance
cDNA: Complementary deoxyribonucleic acid
*Cq*: Quantification cycle
DFd: Degrees of freedom denominator
DFn: Degrees of freedom numerator
DNA: Deoxyribonucleic acid
DPI: Days post-infection
ELISA: Enzyme linked immunosorbent assay
EM: Encysted metacercariae
Foxp3: Forkhead box P3
GAPDH: Glyceraldethde-3-phosphate dehydrogenase
GATA: GATA binding protein
H&E: Hematoxylin and eosin
IFN: Interferon
IL: Interleukin
ITS: Internal transcribed spacer
LSD: Least significant difference
mRNA: Messenger ribonucleic acid
qRT-PCR: Quantitative reverse transcription-polymerase chain reaction
RBC: Red blood cells
rDNA: Ribosomal DNA
RNA: Ribonucleic acid
ROR: Retinoid-related orphan nuclear receptor
RQ: Relative quantitation
SEM: Standard error of the mean
T-bet: T-box expressed in T cells
TGF: Transforming growth factor
Th: T helper
Treg: Regulatory T cell
WPI: Weeks post-infection
